# Molecular Dissection of *Escherichia coli* CpdB: Roles of the N Domain in Catalysis and Phosphate Inhibition, and of the C Domain in Substrate Specificity and Adenosine Inhibition

**DOI:** 10.3390/ijms22041977

**Published:** 2021-02-17

**Authors:** Iralis López-Villamizar, Alicia Cabezas, Rosa María Pinto, José Canales, João Meireles Ribeiro, Joaquim Rui Rodrigues, María Jesús Costas, José Carlos Cameselle

**Affiliations:** 1 Grupo de Enzimología, Departamento de Bioquímica y Biología Molecular y Genética, Facultad de Medicina, Universidad de Extremadura, 06006 Badajoz, Spain; iralis80@hotmail.com (I.L.-V.); acabezas@unex.es (A.C.); rospinto@unex.es (R.M.P.); canales@unex.es (J.C.); jribeiro@unex.es (J.M.R.); macostas@unex.es (M.J.C.); 2 Laboratório Associado LSRE-LCM, Escola Superior de Tecnologia e Gestão, Instituto Politécnico de Leiria, 2411-901 Leiria, Portugal; joaquim.rodrigues@ipleiria.pt

**Keywords:** pathogen–host interaction, cyclic dinucleotide, extracytoplasmic phosphodiesterase, protein domain, truncated protein, point mutant, substrate-binding site, catalytic site, substrate specificity, inhibitor sensitivity

## Abstract

CpdB is a 3′-nucleotidase/2′3′-cyclic nucleotide phosphodiesterase, active also with reasonable efficiency on cyclic dinucleotides like c-di-AMP (3′,5′-cyclic diadenosine monophosphate) and c-di-GMP (3′,5′-cyclic diadenosine monophosphate). These are regulators of bacterial physiology, but are also pathogen-associated molecular patterns recognized by STING to induce IFN-β response in infected hosts. The *cpdB* gene of Gram-negative and its homologs of gram-positive bacteria are virulence factors. Their protein products are extracytoplasmic enzymes (either periplasmic or cell–wall anchored) and can hydrolyze extracellular cyclic dinucleotides, thus reducing the innate immune responses of infected hosts. This makes CpdB(-like) enzymes potential targets for novel therapeutic strategies in infectious diseases, bringing about the necessity to gain insight into the molecular bases of their catalytic behavior. We have dissected the two-domain structure of *Escherichia coli* CpdB to study the role of its N-terminal and C-terminal domains (CpdB_Ndom and CpdB_Cdom). The specificity, kinetics and inhibitor sensitivity of point mutants of CpdB, and truncated proteins CpdB_Ndom and CpdB_Cdom were investigated. CpdB_Ndom contains the catalytic site, is inhibited by phosphate but not by adenosine, while CpdB_Cdom is inactive but contains a substrate-binding site that determines substrate specificity and adenosine inhibition of CpdB. Among CpdB substrates, 3′-AMP, cyclic dinucleotides and linear dinucleotides are strongly dependent on the CpdB_Cdom binding site for activity, as the isolated CpdB_Ndom showed much-diminished activity on them. In contrast, 2′,3′-cyclic mononucleotides and bis-4-nitrophenylphosphate were actively hydrolyzed by CpdB_Ndom, indicating that they are rather independent of the CpdB_Cdom binding site.

## 1. Introduction

CpdB, the product of the *cpdB* gene of *Escherichia coli*, was originally identified as a periplasmic enzyme with phosphohydrolytic activity on 2′,3′-cyclic mononucleotides and 3′-nucleotides [1,2,3]. When studied as a recombinant protein, besides showing these activities at a very high catalytic efficiency, CpdB acted also as phosphodiesterase of cyclic dinucleotides, with an efficiency acknowledged to be comparable to other c-di-AMP (3′,5′-cyclic diadenosine monophosphate) or c-di-GMP (3′,5′-cyclic diguanosine monophosphate) phosphodiesterases [4,5]. Cyclic dinucleotide phosphodiesterases include protein (sub)classes characterized by different domain combinations: c-di-GMP-preferring phosphodiesterases bear either an EAL or a HD-GYP domain, and c-di-AMP-preferring phosphodiesterases bear either a HD domain bound to a 7TM receptor or both DHH and DHHA1 domains (reviewed in [5]). CpdB does not belong to any of these protein classes. According to the Conserved Domains database [6], it contains a N-terminal metallophos or calcineurin-like phosphoesterase domain (Pfam ID PF00149), typical of the metallophosphoesterases or “metallodependent phosphatases” superfamily (SCOP2 ID 3001067), and a 5′-nucleotidase, C-terminal domain (5_nucleotid_C; PF02872). In addition, the periplasmic location of CpdB makes it fit to hydrolyze extracellular cyclic dinucleotides, similarly to the cell wall-anchored CdnP phosphodiesterase of group B *Streptococcus* [7] (see below). The extracytoplasmic location of CpdB and CdnP enzymes is in interesting contrast to the cytoplasmic character of EAL, HD or DHH domain cyclic dinucleotide phosphodiesterases, which modulate bacterial physiology by hydrolysis of intracellular substrates.

Concerning the biological role of CpdB, two aspects are worth considering. On the one hand, it plays in the economy of phosphate through the scavenging of nucleotidic derivatives like 2′,3′-cyclic mononucleotides and their 3′-nucleotide hydrolytic products, compounds of uncertain origin except by the possibility that they are formed by ribonuclease degradation of extracytoplasmic RNA [8,9,10]. On the other hand, there is consistent evidence for a role in virulence of the *cpdB* gene of gram-negative bacteria encoding periplasmic enzyme [11,12], and of a *cpdB*-like gene of gram-positive bacteria, encoding cell wall-anchored enzyme, and named *sntA* in *Streptococcus suis* or *cdnP* in *S. agalactiae* [7,13,14,15,16,17]. One should also be aware of the occurrence of another pro-virulent cyclic nucleotide phosphodiesterase of *Mycobacterium tuberculosis*, a DHH-DHHA1 domain protein encoded by the *Rv2837c* gene and named CdnP, although it is not homologous to *S. agalactiae* CdnP or to *E. coli* CpdB, and its outward/inward orientation in *M. tuberculosis* membrane is unclear [18,19,20]. Pro-virulent effects of CpdB(-like) enzymes are difficult to rationalize in terms of phosphohydrolase activities over 2′,3′-cyclic mononucleotides and 3′-nucleotides, but they can be explained by the hydrolysis of cyclic dinucleotides in the bacterial extracytoplasmic compartment [4,7], since these regulators are pathogen-associated molecular patterns (PAMP) that trigger innate immunity in the infected host [21,22,23,24,25,26,27,28]. In fact, it has been shown that CdnP of the extracellular pathogen *S. agalactiae* (able to survive in macrophage phagosomes) dampens the production of type I interferon by hydrolyzing c-di-AMP secreted by the pathogen [7]. The occurrence of this enzymatic stratagem that interferes with innate immunity mechanisms points to these enzymes as targets to develop novel therapeutic strategies in infectious diseases [7,19,20,29,30]. Therefore, it is imperative to gain insight into the molecular bases of their catalytic behavior.

There is no crystal structure for CpdB, but its domain architecture is similar to that of the archetypical 5′-nucleotidase UshA, although CpdB is a highly efficient 3′-nucleotidase devoid of 5′-nucleotidase activity [4]. UshA is a 5′-nucleotidase and UDP-sugar hydrolase [31,32,33,34,35], also active on diadenosine-polyphosphates [36] and CDP-alcohols [37], but it is essentially inactive on 3′-nucleotides. Structural studies of UshA reveal two domains with different functions: an N-terminal domain, with a dimetallic center that is part of the catalytic site of the enzyme, and a C-terminal domain with a substrate-binding pocket. During catalysis, nucleotidic substrates bind to the specificity site while UshA is in an open conformation. After a large hinge-bending C-domain rotation, the terminal phosphate group of the substrate enters the dimetal catalytic pocket [38,39,40,41,42,43,44,45]. Based on the similar domain architecture of CpdB and UshA, the catalytic cycle of CpdB could also be similar to that of UshA. Considering the recent interest on CpdB(-like) pro-virulent enzymes, we decided to investigate this hypothesis by preparing molecular models of *E. coli* CpdB, and by constructing point mutants of CpdB, and truncated proteins corresponding to the N-terminal and C-terminal domains (CpdB_Ndom and CpdB_Cdom), to study their substrate and inhibitor specificities.

## 2. Results

### 2.1. Molecular Modeling of CpdB Reveals a Two-Domain Conformation with Overall Similitude to 5′-Nucleotidase UshA and with Sequential and Spatial Conservation of Catalytic Histidine and Substrate-Binding Aromatic Residues

**Figure 1:**
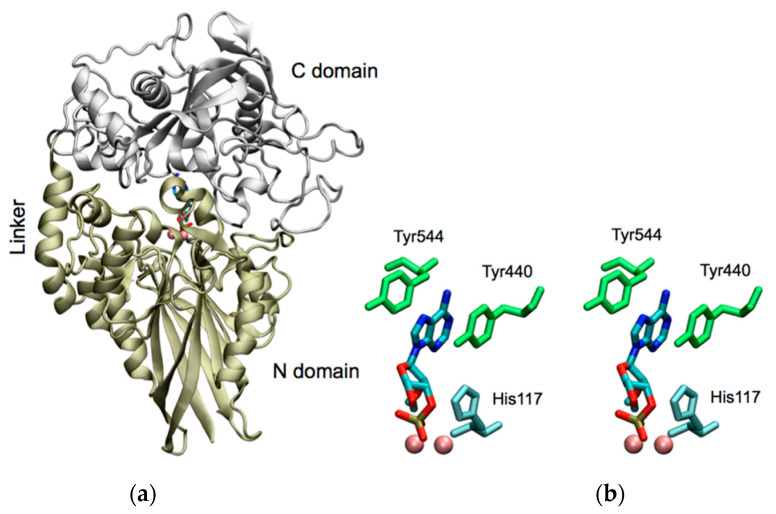
Structure of mature CpdB from *E. coli* with docked 3′-AMP. (**a**) Homology model. The N-terminal domain (colored in tan, including the linker, residues 20–354) adopted the structure of a metallophos or calcineurin-like phosphoesterase domain, typical of the dimetallic metallophosphoesterases or “metallodependent phosphatases” superfamily. Metal ions are colored in pink. The C-terminal domain (colored in silver; residues 355–647) adopted the 5_nucleotid_C structure that fits into the 5′-nucleotidase family proteins. The model shows CpdB in a closed conformation with 3′-AMP bound in the cleft between the metallophos and the 5_nucleotid_C domains. (**b**) Stereogram of 3′-AMP (colored by atom) docked to the active site in the closed conformation. Tyr^440^ and Tyr^544^ residues (green) belong to the C domain; His^117^ (cyan) and the dinuclear metallic center belong to the N domain.

A model of mature CpdB, i.e., the precursor protein devoid of the 19-amino acid signal sequence typical of periplasmic proteins of Gram-negative bacteria, was constructed by homology. It displays N- and C-terminal domains bound by a 20-amino acid linker in a closed conformation (Figure 1a). The N domain displays the features of the metallophosphoesterases, including a dinuclear metallic center typical of the catalytic site of this protein superfamily [46]. The overall CpdB structure resembles that of the 5′-nucleotidase UshA, while they share 24% of amino acid identity (Figure S1). Central features of the catalytic cycle of UshA are the hydrophobic stacking of the purine ring of the substrate with Phe^429^ and Phe^498^, and the introduction of a terminal phosphoryl group of the substrate in the dimetal catalytic pocket where His^117^ plays an essential catalytic role [39]. Therefore, the main aspects of the CpdB model to be considered are: the overall resemblance to UshA, the sequential conservation of His^117^ in both proteins, the sequential conservative substitution of UshA Phe^429^ and Phe^498^ by CpdB Tyr^440^ and Tyr^544^ (Figure S1), and the spatial conservation of the histidine and the aromatic residues. In fact, a docking simulation of 3′-AMP to the CpdB model of Figure 1a indicated that this major substrate can fit with its phosphate group near the dimetal center and the catalytic histidine of the N domain, while the adenine ring is inserted between the two tyrosine residues of the C domain (Figure 1b), similarly to the fit of a 5′-nucleotide analog in the closed conformation of UshA [39].

### 2.2. Catalytic Properties of the N and C Domains of Mature CpdB Expressed Separately, and of Point Mutants of Mature CpdB: His^117^Ala in the Catalytic Site (N Domain) and Tyr^544^Ala in the Substrate Binding Site (C Domain)

Mature CpdB has been recently expressed and its catalytic behavior was studied with a large series of substrates [4]. In the current work, to dissect the roles of the N and C domains of CpdB, they were constructed and expressed separately (CpdB_Ndom and CpdB_Cdom). First, it must be remarked that CpdB_Cdom was inactive on any tested substrate, which agrees with the model in that it does not contain the catalytic site. On the other hand, CpdB_Ndom was catalytically active but the absence of the C domain affected differentially the activities on diverse substrates.

**Figure 2:**
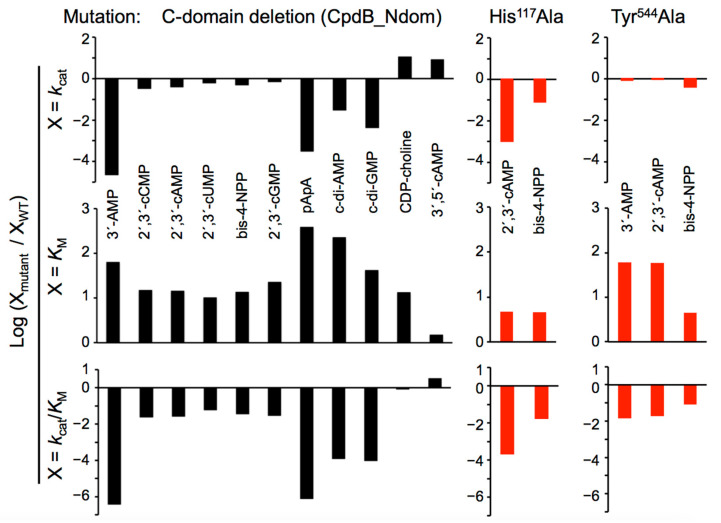
Effect of mutations on the kinetic parameters of CpdB. The mean values of the catalytic parameters of Table 1 were divided by the corresponding values of wild-type CpdB as determined in earlier work [4] and shown also in Table 1. Substrates giving insignificant rates with the point mutants (see footnotes 2 and 3 in Table 1) cannot be plotted here as in the absence of significant rates, kinetic parameters cannot be estimated.

In the first evaluation of CpdB_Ndom activities on the substrates of the mature CpdB, hydrolytic rates at a fixed 750 µM substrate concentration confirmed the absence of detectable activity on 5′-AMP and 2′-AMP, like is the case of mature CpdB, and the almost complete loss of activities (anyhow detectable at relatively high concentrations of CpdB_Ndom) on 3′-AMP and cyclic or linear dinucleotides. Nevertheless, CpdB_Ndom conserved noticeable activities on other substrates, mainly 2′,3′-cyclic mononucleotides and bis-4-nitrophenylphosphate (bis-4-NPP). With all the detectably hydrolyzed substrates, saturation kinetics was studied and parameters *k*_cat_ and *K*_M_ were determined from Michaelis-Menten plots (Table 1). The effect of deleting the C domain of CpdB in the kinetic parameters is summarized in Figure 2. Concerning *k*_cat_ values, three groups of substrates were clearly distinguished: 3′-AMP and cyclic or linear dinucleotides displayed strong activity losses (30–44000 fold); 2′,3′-cyclic mononucleotides and bis-4-NPP showed only small losses (1.4–3 fold); the minor CpdB substrates CDP-choline and 3′,5′-cAMP displayed small but clear *k*_cat_ increases (8–11 fold). A clearcut increase of *K*_M_ value (10–400 fold) relative to CpdB was observed with all the substrates of CpdB_Ndom except with the minor substrate 3′,5′-cAMP. The increase was larger (40–400 fold) for 3′-AMP and cyclic or linear dinucleotides, and only about 10–20 fold for 2′,3′-cyclic mononucleotides, bis-4-NPP and CDP-choline.

The role of the possible catalytic and substrate-binding sites was additionally tested by the preparation of two point mutants of the mature CpdB protein. On the one hand, the mutation His^117^Ala modified the histidine residue conserved in the N domain (Figure 1b) with respect to UshA, where it has been proposed to be part of the catalytic core structure and to act as a general base that facilitates the nucleophilic attack by deprotonating a water molecule, and to participate in the stabilization of the transition state [39]. The His^117^Ala mutant of CpdB was essentially inactive on 3′-AMP and the cyclic and linear dinucleotides, but conserved minor activity on 2′,3′-cAMP and the artificial phosphodiester bis-4-NPP, with catalytic efficiencies (*k*_cat_/*K*_M_) 5000-fold and 60-fold lower, respectively, than wild-type mature CpdB for the same substrates. On the other hand, mutation Tyr^544^Ala modified one of the aromatic residues of CpdB which are conserved in the C domain with respect to the UshA phenylalanine residues that form a stacked sandwich with the adenine ring of the substrate [39]. This disposition was confirmed by docking of 3′-AMP to the structural model of CpdB (Figure 1). The Tyr^544^Ala mutant conserved substantial catalytic activity on 3′-AMP (*k*_cat_ 160 ± 37 s^−1^), 2′,3′-cAMP (*k*_cat_ 190 ± 60 s^−1^) and bis-4-NPP (*k*_cat_ 130 ± 34 s^−1^) similar or only somewhat lower than wild-type mature CpdB for the same substrates. However, the *K*_M_ values were increased by the Tyr^544^Ala mutation: 60 fold for 3′-AMP and 2′,3′-cAMP, and only five-fold for bis-4-NPP. According to these kinetic parameters, the Tyr^544^Ala substitution diminished the catalytic efficiencies for these substrates from 10^6^–10^7^ M^−1^s^−1^ to 1.4–3 × 10^5^ M^−1^s^−1^. Concerning the activities on 2′,3′-cAMP and bis-4-NPP, the Tyr^544^Ala mutation practically mimicked the deletion of the C domain. However, this was not so for 3′-AMP, as the catalytic efficiency on this substrate was ≈ 2 × 10^5^ M^−1^s^−1^ with the point mutant and only 5 M^−1^s^−1^ with CpdB_Ndom.

### 2.3. Alteration of the Reaction Products of the Hydrolysis of Cyclic (di)Nucleotides by the Deletion of the C Domain of Mature CpdB

**Figure 3:**
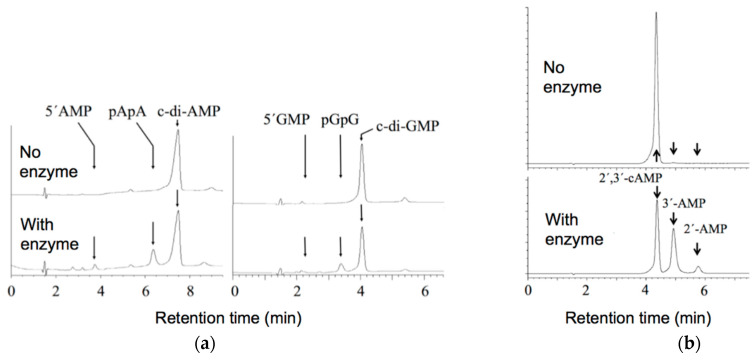
Products of the hydrolysis of cyclic (di)nucleotides by CpdB_Ndom. (**a**) Hydrolysis of c-di-AMP or c-di-GMP at 20 µM concentration of the dinucleotide. (**b**) Hydrolysis of 2′,3′-cAMP at 500 µM. The traces were recorded at 260 nm. Arrows indicate the retention times of the standard compounds. pGpG, 5′-phosphoguanylyl-3′→5′-guanosine.

The hydrolysis of c-di-AMP and c-di-GMP by mature CpdB is known to yield AMP or GMP as the very major, or even the only detectable product, which has been interpreted as the consequence of the consecutive hydrolysis of the two phosphodiester linkages of those substrates [4]. In contrast, the hydrolysis of the same substrates by CpdB_Ndom yielded the linear dinucleotides, pApA (5′-phosphoadenylyl-3′→5′-adenosine) or pGpG (5′-phosphoguanylyl-3′→5′-guanosine), as the major products with only minor amounts of 5′-nucleotides, AMP or GMP, detected at least in the first phase of the reactions (Figure 3a). In this concern, the difference between mature CpdB and CpdB_Ndom is in good correlation with the 100-fold stronger loss of catalytic efficiency on pApA than on c-di-AMP caused by the deletion of the C domain (Figure 2).

The hydrolysis of 2′,3′-cAMP by mature CpdB yields 3′-AMP which is immediately hydrolyzed to adenosine and phosphate, without any indication of the formation of 2′-AMP [4]. Instead, the hydrolysis of 2′,3′-cAMP by CpdB_Ndom yielded 3′-AMP as the major product (85%), without conversion to adenosine, but accompanied by a 15% of 2′-AMP (Figure 3b). The accumulation of 3′-AMP rather than adenosine can be explained by the 10^5^-fold stronger loss of catalytic efficiency on 3′-AMP than on 2′,3′-cAMP caused by the deletion of the C domain (Figure 2). The formation of a certain proportion of 2′-AMP by CpdB_Ndom cannot be explained by a loss of activity on 2′-AMP upon C-domain deletion, because mature CpdB lacks this activity [4]. Hence, the partial conversion of 2′,3′-cAMP to 2′-AMP by CpdB-Ndom should be attributed to a less specific orientation of 2′,3′-cAMP in the catalytic site in the absence of the substrate-binding C domain.

### 2.4. Inhibition of the Activity of Mature CpdB and CpdB_Ndom on 3′-AMP or bis-4-NPP by Alternative Substrates and Non-Substrates

**Figure 4:**
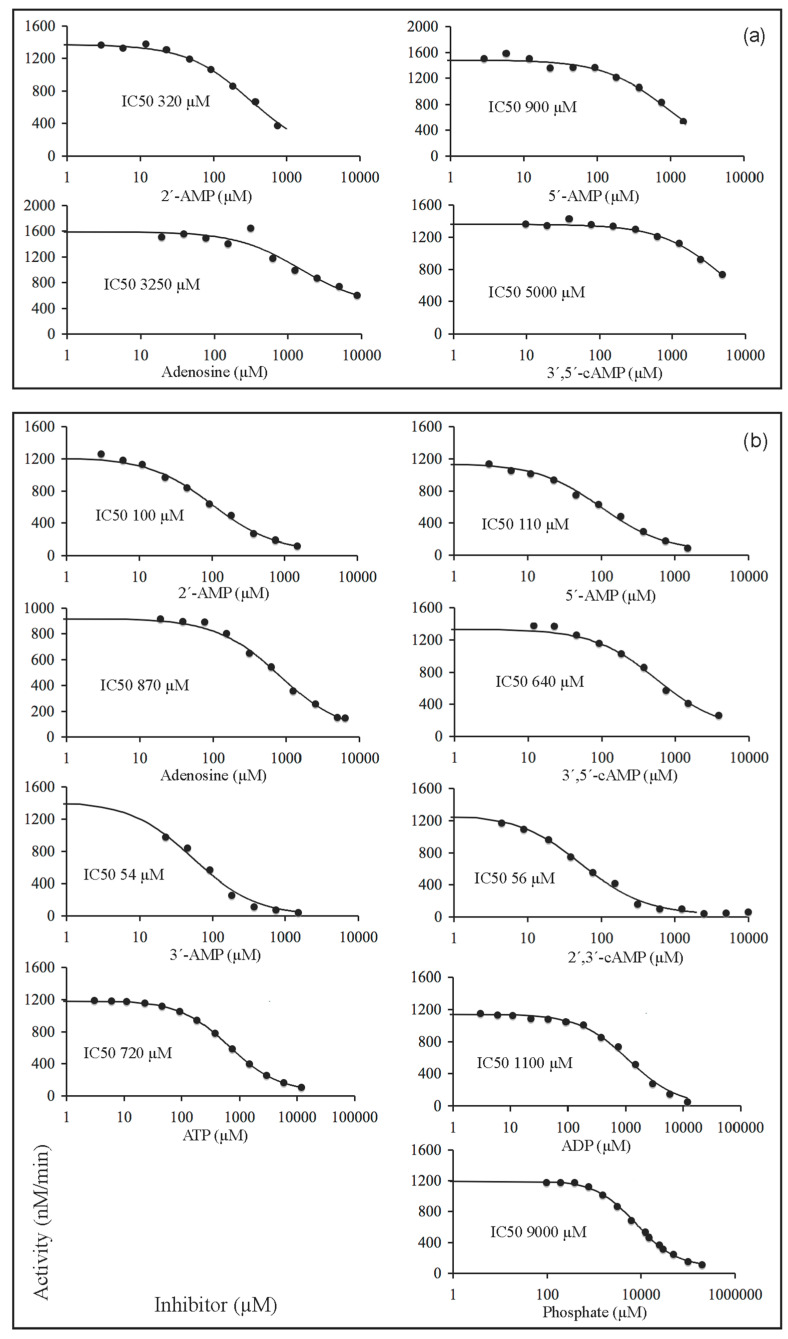
Inhibitor dose-response curves of mature CpdB. IC50 is the inhibitor concentration causing 50% inhibition for each particular substrate-inhibitor combination. The panels are single illustrative experiments. (**a**) Curves were recorded with 370 µM 3′-AMP as the substrate. (**b**) Curves were recorded with 750 µM bis-4-NPP as the substrate. The curves were obtained by fitting the equation for a mixed partial inhibition to the experimental data, as in Figure 5.

**Figure 5:**
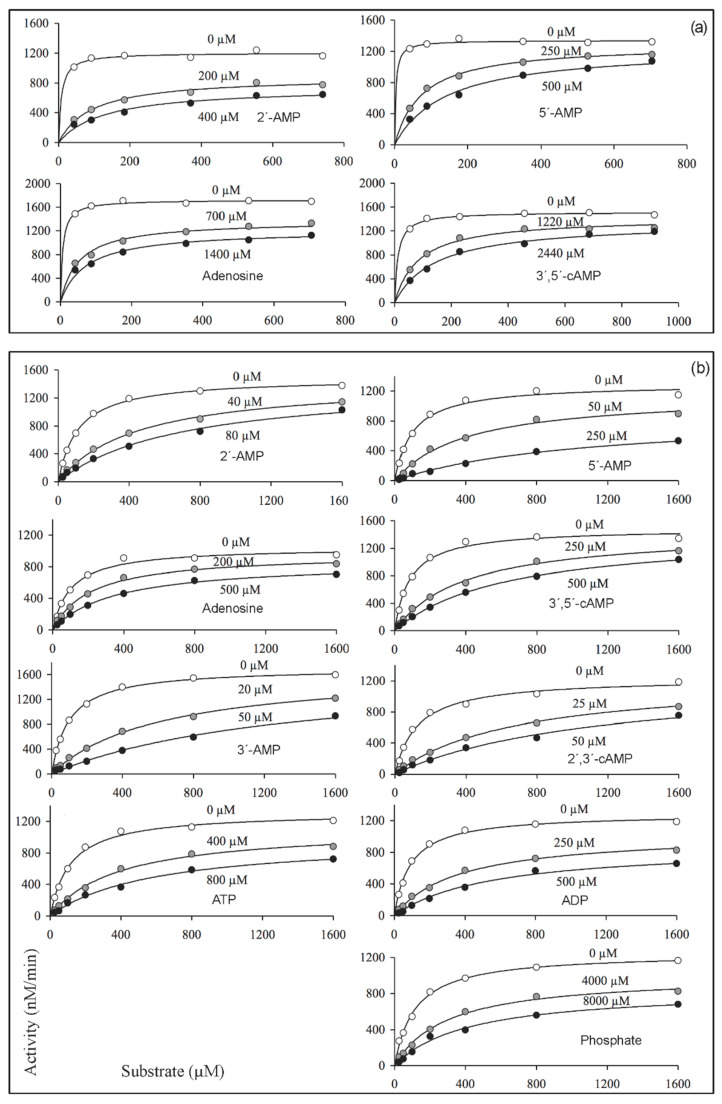
Kinetic analysis of the inhibition of mature CpdB by alternative substrates and non-substrates of the enzyme. Saturation curves by 3′-AMP (**a**) or bis-4-NPP (**b**) were recorded in the absence or in the presence of two fixed concentrations of the compound tested as an inhibitor (data indicated in each panel). The three curves of each panel were obtained by globally fitting the equation for a mixed partial inhibition to all the experimental data. Each panel is representative of three independent experiments, except panels in (**b**) with 5′-AMP, adenosine, 3′,5′-cAMP, and ADP as inhibitors which represent single experiments. The inhibition constants (*K*_i_) estimated after these adjustments are summarized in Table 2.

In search of possible ways to differentiate between the putative substrate-binding site and the catalytic site of CpdB, we decided to perform inhibitor studies and to compare their effects on mature CpdB and CpdB_Ndom. In this approach, inhibitors were used as probes for the relevance of those enzyme sites, irrespective of the physiological character and concentrations of the compounds used. Since inhibition experiments were not included in our earlier study of mature CpdB [4], we started now by exploring its inhibition by competing substrates and by non-substrates. For these experiments, we selected two different substrates: 3′-AMP, which is the one showing the highest catalytic efficiency (1.3 × 10^7^ M^−1^s^−1^), and bis-4-NPP, which is somewhat less efficiently hydrolyzed (3.6 × 10^6^ M^−1^s^−1^) but has the important advantage of allowing the use of a direct colorimetric assay of 4-nitrophenol formation by measuring *A*_405_ [4]. With 3′AMP as the substrate, the hydrolysis was assayed by measuring the liberation of phosphate, and the compounds tested as inhibitors were 2′-AMP, 5′-AMP, 3′,5′-cAMP and adenosine. None of them is significantly hydrolyzed by CpdB (to note only the very inefficient hydrolysis of 3′,5′-cAMP, with catalytic efficiency 2.2 × 10^2^ M^−1^s^−1^, i.e., 59,000-fold lower than that of 3′-AMP [4]), so they were not expected to interfere with the measurement of the hydrolysis of 3′-AMP except as activity modifiers. With bis-4-NPP as the substrate, in addition to the same non-substrate compounds tested on the hydrolysis of 3′-AMP, it was also possible to test phosphate and alternative substrates of CpdB as inhibitors, namely 3′-AMP, 2′,3′-cAMP, ATP and ADP, which despite being themselves hydrolyzed do not give any signal in the 4-nitrophenol assay. Altogether, 13 substrate-inhibitor combinations were submitted to two kinds of experiments: a preliminary set of inhibitor dose-response curves at fixed substrate concentration (Figure 4), and substrate saturation curves recorded at different fixed concentrations of inhibitors (Figure 5).

With 3′-AMP as substrate at 370 µM concentration (25-fold higher than its *K*_M_ value [4]), the four weak or non-substrates tested as inhibitors of mature CpdB acted with increasing potencies in the order 2′-AMP > 5′-AMP >> adenosine ≥ 3′,5′-cAMP, with IC50 values ≈300 µM–5000 µM (Figure 4a). With bis-4-NPP as substrate at 750 µM concentration (8-fold higher than its *K*_M_ value [4]) the same compounds elicited stronger inhibitions although with similar relative potencies: 2′-AMP ≥ 5′-AMP >> 3′,5′-cAMP ≥ adenosine (Figure 4b). The stronger effects on bis-4-NPP could be accounted for by the higher fractional saturation with 3′-AMP under the assay conditions.

The five compounds tested only with bis-4-NPP as substrate were four alternative substrates of CpdB (3′-AMP, 2′,3′-cAMP, ATP and ADP) and phosphate. Considering all the inhibitors tested on the hydrolysis of bis-4-NPP, they acted with increasing potencies in the order: 3′-AMP = 2′,3′-cAMP > 2′-AMP ≥ 5′-AMP >> 3′,5′-cAMP ≥ ATP ≥ adenosine ≥ ADP >> phosphate (Figure 4b).

The type of inhibition produced in each of the thirteen substrate-inhibitor combinations tested was determined in substrate saturation experiments run, with 3′-AMP or bis-4-NPP as the substrate, in the absence and in the presence of two different inhibitor concentrations (Figure 5). The results were analyzed by globally fitting to the data points the kinetic equations corresponding to competitive, mixed (general non-competitive) and uncompetitive inhibition, either complete or partial [47,48,49]. In every case, the best fit was obtained with the equation for the partial, mixed type of inhibition, which yielded the curves shown in Figure 5. The dissociation constants (*K*_i_) estimated for the binary complexes of mature CpdB with each inhibitor are summarized in Table 2. The relative potencies of inhibition are in good agreement with those estimated from IC50 values derived from dose-response curves (Figure 4). It is worth noting that although a mixed-type inhibition means that the inhibitor may affect both *V*_max_ (*k*_cat_) and *K*_M_, the effect was in every case stronger on *K*_M_ (2–25-fold increase) than on *k*_cat_ (1.0–1.5-fold decrease). So, it can be said that the mixed type inhibition was closer to a competitive inhibition than to a classical non-competitive interaction. In some cases, it could be truly competitive.

Another observation worth noting is that the non-substrates 2′-AMP and 5′-AMP were stronger inhibitors of bis-4-NPP hydrolysis than some CpdB substrates (ATP and ADP) and only a little less strong than the best CpdB substrates 3′-AMP and 2′,3′-cAMP. This, together with the somewhat partial character of the inhibition in some cases, indicates complexity of ligand binding to CpdB, with the possible occurrence of more than one binding site.

**Figure 6:**
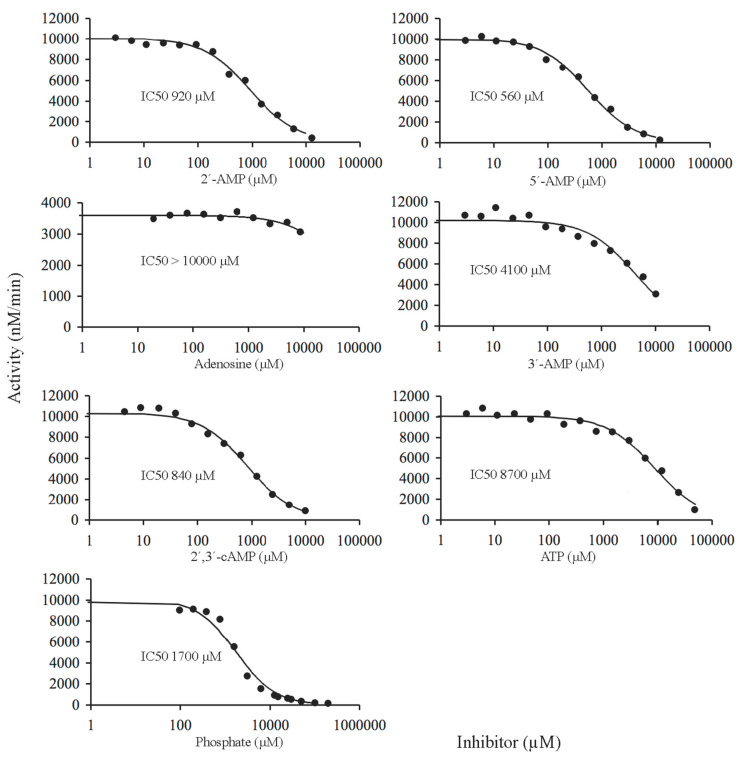
Inhibitor dose-response curves of CpdB_Ndom. IC50 is the inhibitor concentration causing 50% inhibition for each particular substrate-inhibitor combination. All the curves were recorded with 750 µM bis-4-NPP as the substrate. The panels are single illustrative experiments. The curves were obtained by fitting the equation for complete competitive inhibition to the experimental data as in Figure 7.

**Figure 7:**
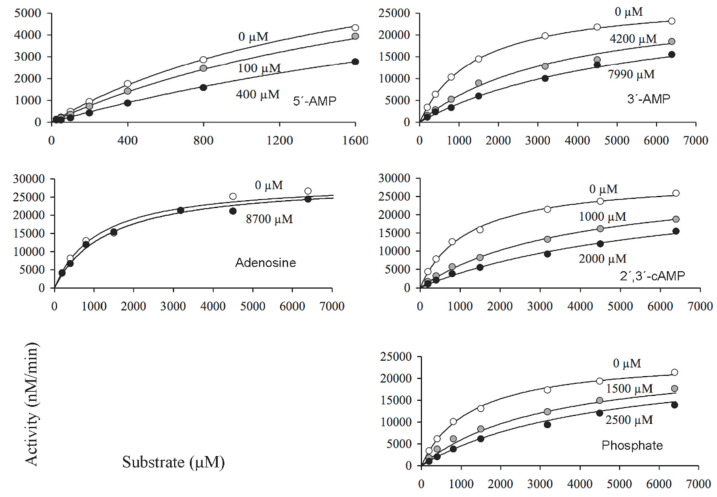
Kinetic analysis of the inhibition of CpdB_Ndom by alternative substrates and non-substrates of the enzyme. Saturation curves by bis-4-NPP were recorded in the absence or in the presence of two (only one for adenosine) fixed concentrations of the compound tested as an inhibitor (data indicated in each panel). The curves of each panel were obtained by globally fitting the equation for complete competitive inhibition to all the experimental data. Each panel shows a representative experiment of three independent ones. The inhibition constants (*K*_i_) estimated after these adjustments are summarized in Table 2.

Concerning the effects of inhibitors on CpdB_Ndom, only the activity on bis-4-NPP was considered because CpdB_Ndom was essentially inactive on 3′-AMP (Table 1). A preliminary set of inhibitor dose-response curves at fixed substrate concentration are shown in Figure 6, where the inhibitors tested display increasing potencies in the order: 5′-AMP ≥ 2′,3′-cAMP ≥ 2′-AMP > phosphate > 3′-AMP > ATP >> adenosine. Substrate saturation experiments were run in the absence and the presence of one of these inhibitors at fixed concentrations (Figure 7). The results were analyzed by globally fitting to the data points, the kinetic equations corresponding to complete and partial, competitive, mixed (general non-competitive) and uncompetitive inhibition [47,48,49]. In every case, good fits were obtained with the equation for the classical (complete) competitive inhibition, which yielded the curves shown in Figure 7. The dissociation constants (*K*_i_) for the binary complexes of CpdB_Ndom with each tested inhibitor are shown in Table 2.

**Figure 8:**
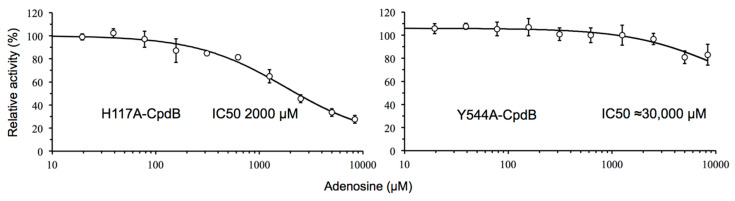
Adenosine dose-response curves of point mutants of CpdB. The curves were recorded with 750 µM bis-4-NPP as the substrate. The data are mean values ± standard deviations of three independent experiments. The curves were obtained by fitting the equation for a mixed partial inhibition to the experimental data.

The two point mutants His^117^Ala and Tyr^544^Ala of mature CpdB were also tested for the inhibition of the hydrolysis of bis-4-NPP by adenosine. Compared to wild–type CpdB, mutation of His^117^ affected little the potency of adenosine inhibition (IC50 increased from 870 µM to 2000 µM), while mutation of Tyr^544^ diminished strongly the potency of inhibition, with IC50 increasing up to an estimated value possibly around 30 mM (Figure 8). The very different effect of both mutations on the potency of adenosine as an inhibitor is in agreement with the locations of the residues submitted to mutation. Tyr^544^ is in the substrate-binding pocket of the C domain and participates in the formation of a stacked sandwich together with Tyr^440^ and the adenine ring of the 3′-AMP substrate (Figure 1b). It seems reasonable to interpret that the removal of the aromatic ring of Tyr^544^ affects negatively the binding of adenosine, while it is neutral towards the hydrolytic activity of CpdB on bis-4NPP (Table 1). On the other hand, the position of His^117^ seems irrelevant to the sandwich Tyr-adenine-Tyr (Figure 1b). It is reasonable thus that His^117^Ala mutation affects little adenosine binding and its inhibitory effect.

## 3. Discussion

The analysis of the conserved domains of CpdB [4] and its molecular modeling (Figure 1) reveal a marked structural similitude with *E. coli* UshA: two domains joined by a flexible linker. Based on this analogy, we hypothesized that the catalytic cycle of CpdB could resemble that of UshA. To test this hypothesis, two types of experiments were performed. For one thing, separate expression and study of the two CpdB domains, and for another, construction and study of two point mutants of the full protein, His^117^Ala which corresponds with the catalytic histidine residue of the UshA active site, and Tyr^544^Ala which corresponds with one of the phenylalanine residues that form a sandwich with the purine base in the UshA substrate-binding pocket. The substrate and inhibitor specificities of the resulting CpdB constructs were in agreement with the hypotheses that the catalytic cycle of CpdB resembles that of UshA in the intervention of separate substrate-binding and catalytic sites, located in different protein domains.

In early studies of CpdB, based solely in the copurification of the 3′-nucleotidase with the 2′,3′-cyclic phosphodiesterase activity, the enzyme was defined as a bifunctional one as long as it displayed phosphomonoesterase and phosphodiesterase activities [1,2,50,51]. From kinetic experiments of mutual inhibition by alternative substrates, and of response to chloride, the occurrence of two partly overlapping active sites was proposed [1,51,52]. Later on, when the *cpdB* gene of *E. coli* was molecularly cloned, Beacham and Garrett set up the hypothesis that, if so, it should be possible to isolate CpdB mutants devoid of one of the activities but not the other [53]. After the expression of CpdB as a recombinant protein [4], the current study of the point mutants His^117^Ala-CpdB and Tyr^544^Ala-CpdB, and of the truncated CpdB_Ndom and CpdB_Cdom (Table 1 and Figure 2) at least in part confirmed this early hypothesis. Indeed, the truncated protein CpdB_Ndom, which contains the catalytic site but not the substrate-binding one, is practically devoid or 3′-nucleotidase activity but keeps a great part of the phosphodiesterase activity on 2′,3′-cyclic mononucleotides and bis-4-NPP. In other words, the activity on 3′-AMP requires both sites, while the activities on 2′,3′-cyclic mononucleotides and bis-4-NPP can dispense with the substrate-binding site.

The differential role of the substrate-binding site and the catalytic one is also evident in the inhibition experiments. If one compares the potency of the inhibition caused by adenosine and its derivatives (either substrates or non-substrates) on the activity of CpdB and CpdB_Ndom with bis-4-NPP as substrate, the absence of the C-terminal domain (CpdB_Ndom) increased the *K*_i_ value 25–1000 fold, but did not change the *K*_i_ value for phosphate (Table 2). Similar differential behavior was observed between the effects of adenosine on the point mutants His^117^Ala-CpdB (inhibition potency similar to wild-type CpdB) and Tyr^544^Ala-CpdB (much weaker inhibition) (Figure 8).

Finally, based on the differential role that the specificity domain plays with different substrates, one arrives at an interesting conclusion. The activities of CpdB on 2′,3′-cyclic mononucleotides and bis-4-NPP, which are near independent of the C-domain substrate-binding site, may be the result of non-specific phosphodiesterase activity, as seen in other phosphohydrolases [54,55]. Nevertheless, this does not mean that the activity on 2′,3′-cyclic mononucleotides is biologically irrelevant. On the other hand, the activities of CpdB on the cyclic dinucleotides c-di-AMP and c-di-GMP, and the linear dinucleotide pApA behaved much like that on 3′-AMP, and require both the catalytic and the substrate-binding sites (Table 1 and Figure 2). Therefore we propose that the molecular architecture of the CpdB protein is specifically designed to act on this group of substrates, which reinforces the view that it may be relevant as cyclic dinucleotide phosphodiesterase in situations in which the *cpdB* gene has been described as a virulence factor [11,12].

## 4. Materials and Methods

### 4.1. Homology Modeling of CpdB and Docking of 3′-AMP to the Active Site

A structural model of mature CpdB (aa 20–647) was built with Modeller 9.5 (https://salilab.org/modeller) (accessed on 16 February 2021) [56] using two PDB (Protein Data Bank)-deposited structures as templates: 3JYF, the *N*-terminal domain (aa 16-354) of a *Klebsiella pneumoniae* protein (UniProt ID A6THC4; length, 647 aa) that shows an 82% identity (88% similar residues) with CpdB, and 4Q7F, a protein from *Staphylococcus aureus* (UniProt ID Q2G1L5; length, 511 aa) that shows homology with aa 30–549 of CpdB (26% identity; 42% similar residues) co-crystalized with 2′-deoxyadenosine in the putative active site. A total of 100 conformations were generated and ranked by a DOPE score [57]. The best one was further prepared for docking experiments. Metals were modeled as Mg^2+^. The program Reduce (http://kinemage.biochem.duke.edu/software/reduce.php) (accessed on 16 February 2021) [58] was used to add hydrogens and to establish the most likely protonation state of histidines in the selected model.

The tridimensional model of 3′-AMP was generated with Marvin (ChemAxon, Budapest, Hungary; https://chemaxon.com) (accessed on 16 February 2021). The receptor and the ligand were prepared with AutoDock Tools [59]. Docking calculations were performed with AutoDock 4 (http://autodock.scripps.edu) (accessed on 16 February 2021) [60] in a search box generated with Autogrid encompassing the putative binding site. A population of 100 conformations was obtained with a maximum of 25 × 10^6^ energy evaluations per conformation. The best conformation (low energy) with 3′-AMP in a position similar to that occupied by 2′-deoxyadenosine in 4Q7F was selected.

### 4.2. Construction of Plasmids Encoding Point Mutants, the N Domain (CpdB_Ndom) and the C Domain (CpdB_Cdom) of the CpdB Protein

These constructs were prepared from plasmid pGEX-6P-3-cpdB, which contains the coding sequence of mature CpdB (GenBank accession KP938772; CpdB precursor without the 19-residue N-terminal signal sequence) inserted between the BamHI and EcoRI sites of pGEX-6P-3 [4]. Plasmids pGEX-6P-3-H117A-cpdB and pGEX-6P-3-Y544A-cpdB, encoding point mutants of CpdB, were prepared following the QuikChange protocol (Stratagene) using the mutagenic primers described in Table S1. The sequence coding for CpdB_Ndom (nucleotides 58–1005 of accession KP938772 with an added stop codon) was amplified using the primers CpdB-Fow and CpdB_Ndom-Rev_a (Table S1). The sequence coding for CpdB_Cdom (nucleotides 1006–1944 of accession KP938772, ending with the natural stop codon of the full protein) was amplified using the primers CpdB_Cdom-Fow_a and CpdB-Rev (Table S1). The two forward primers (Fow) were designed to include BamHI sites immediately before the coding sequence, and the two reverse ones (Rev) were designed such that their reverse complements contained an EcoRI site immediately after a stop codon. The resulting amplicons encode respectively CpdB_Ndom (amino acids 20–354 of the CpdB precursor) and CpdB_Cdom (amino acids 355–647 of the CpdB precursor). Each amplicon was purified and inserted between the BamHI and EcoRI sites of pGEX-6P-3, to obtain plasmids pGEX-6P-3-cpdB_Ndom and pGEX-6P-3-cpdB_Cdom. Double-strand sequencing of the full passengers of all constructions confirmed the presence of the expected coding sequences in frame with the glutathione S-transferase (GST) tag of the pGEX-6P-3 vector (Figures S2 and S3).

### 4.3. Expression and Purification of Recombinant Proteins

Mature CpdB, its point mutants His^117^Ala-CpdB and Tyr^544^Ala-CpdB, CpdB_Ndom and CpdB_Cdom were obtained as earlier described for mature CpdB [4]. In brief, BL21 *E.coli* cells were transformed with the constructed plasmids (Section 4.2), ampicillin-resistant colonies were picked, amplified in culture, and the *tac* promoter-driven expression of the GST fusion proteins encoded by the plasmids was induced with isopropylthiogalactoside (IPTG). The fusion proteins are expressed in the cytoplasm of BL21 cells. After bacterial lysis, the GST fusions were recovered from the supernatants and were purified by affinity chromatography in GSH-Sepharose columns, where they remained adsorbed. The CpdB moieties of the fusion proteins were separated from the GST tag by in-column proteolysis with Prescission protease, which left a GPLGS extension in the N end of CpdB and derivative proteins. Their purity was checked by SDS-PAGE (sodium dodecyl sulfate polyacrylamide gel electrophoresis). The gels were stained with Coomassie Blue and quantified by image analysis with GelAnalyzer 2010 [61] (Figure S4). Final purities were as follows: mature CpdB, 88%; CpdB_Ndom, 96%; CpdB_Cdom, 83%; His^117^Ala-CpdB, 98%; Tyr^544^Ala-CpdB, 100%. Protein content was assayed according to Bradford [62].

### 4.4. Enzyme Activity Assays

The procedures and detailed conditions to measure the initial rates of phosphohydrolytic reactions were as earlier described [4]. The hydrolysis of 3′-AMP was assayed by measuring the direct liberation of inorganic phosphate by a sensitive colorimetric assay. The same assay was used with 2′,3′- or 3′,5′-cyclic nucleotides, CDP-choline and bis-4-NPP except that an excess of alkaline phosphatase was included in the reaction mixture as auxiliary enzyme. The hydrolysis of linear or cyclic dinucleotides, and sometimes 2′,3′-cAMP, were assayed by HPLC (high-performance liquid chromatography) measuring the accumulation of 5′-AMP, 5′-GMP 3′-AMP or 2′-AMP as products.

To study the inhibition of the hydrolysis of bis-4-NPP by different compounds, the reaction rate was assayed by measuring colorimetrically the formation of 4-nitrophenol by its absorbance at 405 nm. Reaction mixtures of 100 µL contained, besides the needed amount of enzyme (i.e., 0.02–0.05 µg/mL of mature CpdB, 2 µg/mL of a CpdB point mutant, or 0.2 µg/mL of CpdB_Ndom), 50 mM Tris [Tris(hydroxymethyl)aminomethane]-HCl pH 7.5 (adjusted at 37 °C), 2 mM MnCl_2_, 0.1 mg/mL bovine serum albumin, and the indicated concentrations of bis-4-NPP and the compound tested as inhibitor. Incubations performed at 37 °C were stopped by addition of 0.5 mL of 200 mM Tris, pH 8.9, 5 mM EDTA (ethylenediaminetetraacetate), immediately followed by *A*_405_ measurement (ε = 18.1 mM^−1^ cm^−1^). This termination procedure was used instead of the addition of a high NaOH concentration, to avoid the known formation of brown precipitates of air-oxidized Mn^2+^ hydroxide. On occasions, when a higher sensitivity was needed, reaction mixtures of 500 µL were implemented and termination was performed with 0.1 mL 1M Tris, pH 8.9, 30 mM EDTA.

### 4.5. Analyses of Saturation and Inhibition Kinetics

The procedures followed to derive kinetic parameters *k*_cat_, *K*_M_ and *K*_i_ from initial rate vs. substrate concentration datasets, either in the presence or the absence of inhibitor, were as described elsewhere [49]. Datasets obtained with different substrates in the absence of inhibitors were used to derive *k*_cat_ and *K*_M_ values by adjusting the Michaelis-Menten equation to the experimental points by non-linear regression. On the other hand, substrate saturation datasets obtained in the presence of three different inhibitor concentrations (one of them null), were used to find the best fitting kinetic equation out of six possibilities, i.e., complete or partial, competitive, mixed or uncompetitive inhibition [47,48]. Each one of these equations was simultaneously adjusted (global fit) by nonlinear regression to the three saturation curves of each inhibition experiment, using the Solver function of Microsoft Excel. During the adjustments, *V*_max_*, K*_M_ and *K*_i_ were left to fluctuate. The precision of fit was estimated with a χ^2^ parameter, with lower values indicating better fits [48,49]. In each set of experiments, the type of inhibition that gave the best fit was used as the model to estimate *K*_i_ values.

## Figures and Tables

**Table 1:** Kinetic parameters of the N-terminal domain of CpdB (CpdB_Ndom) and point mutants of mature CpdB. Results are expressed as mean values ± standard deviations of three independent experiments.

Protein	Substrate	*k*_cat_	*K*_M_	*k*_cat_ */K*_M_
		s^−1^	µM	M^−1^ s^−1^
CpdB_Ndom ^1^	2′,3′-cUMP	155 ± 32	630 ± 170	250,000 ± 50,000
	2′,3′-cCMP	33 ± 5	180 ± 50	200,000 ± 80,000
	2′,3′-cAMP	73 ± 32	390 ± 170	190,000 ± 13,000
	bis-4-NPP	164 ± 14	1300 ± 80	130,000 ± 7000
	2′,3′-cGMP	58 ± 16	570 ± 160	100,000 ± 13,000
	CDP-choline	5.7 ± 3.5	2900 ± 1700	1950 ± 130
	3′,5′-cAMP	4.7 ± 1.6	6900 ± 2600	700 ± 60
	3′-AMP	0.004 ± 0.0002	880 ± 250	5.2 ± 1.0
	c-di-AMP	0.012 ± 0.007	3400 ± 2200	3.5 ± 0.1
	pApA	0.0004 ± 0.0001	230 ± 40	1.8 ± 0.2
	c-di-GMP	0.0003 ± 0.0001	250 ± 30	1.2 ± 0.4
His^117^Ala-CpdB ^2^	bis-4-NPP	26 ± 3	440 ± 90	60,000 ± 7000
	2′,3′-cAMP	0.18 ± 0.02	126 ± 7	1500 ± 230
Tyr^544^Ala-CpdB ^3^	bis-4-NPP	130 ± 34	430 ± 30	300,000 ± 100,000
	3′-AMP	160 ± 37	850 ± 110	190,000 ± 62,000
	2′,3′-cAMP	190 ± 60	1600 ± 550	140,000 ± 73,000
Mature CpdB ^4^	2′,3′-cUMP	260	62	4,200,000
	2′,3′-cCMP	100	12	8,500,000
	2′,3′-cAMP	190	27	7,300,000
	bis-4-NPP	340	96	3,600,000
	2′,3′-cGMP	84	25	3,500,000
	CDP-choline	0.51	219	2300
	3′,5′-cAMP	0.56	4600	220
	3′-AMP	176	14	13,000,000
	c-di-AMP	0.40	15	29,000
	pApA	1.30	0.6	2,300,000
	c-di-GMP	0.07	6	13,000

^1^ The C-terminal domain of CpdB (CpdB_Cdom) was inactive with all the substrates tested. ^2^ Essentially inactive on 3′-AMP and cyclic or linear dinucleotides. ^3^ Essentially inactive on cyclic or linear dinucleotides. ^4^ Data taken from previous work [4] and shown here to facilitate comparison with mutant proteins. Bis-4-NPP, bis-4-nitrophenylphosphate; c-di-AMP, 3′,5′-cyclic diadenosine monophosphate; c-di-GMP, 3′,5′-cyclic diguanosine monophosphate; pApA, 5′-phosphoadenylyl-3′→5′-adenosine.

**Table 2:** Inhibition constants (*K*_i_) of alternative substrates and non-substrates of mature CpdB and CpdB_Ndom. The values were obtained during the analyses of substrate saturation curves with(out) inhibitor by the global fit of the equation to the experimental data. Whenever the results are shown as mean values ± standard deviations, they correspond to three independent experiments.

Substrate	Inhibitor	CpdB ^1^	CpdB_Ndom ^2^
		µM	µM
3′-AMP	2′-AMP	7.1 ± 3.2	—
	5′-AMP	20 ± 8	—
	Adenosine	41 ± 17	—
	3′,5′-cAMP	145 ± 38	—
Bis-4-NPP	2′-AMP	8.8 ± 2.0	600 ^3^
	5′-AMP	14	370 ± 50
	Adenosine	160	48,000 ± 11,000
	3′,5′-cAMP	60	—
	3′-AMP	3.6 ± 0.2	3300 ± 550
	2′,3′-cAMP	5.0 ± 0.4	460 ± 50
	ATP	113 ± 19	5700 ^3^
	ADP	60	—
	Phosphate	1380 ± 340	1060 ± 210

—, not determined. ^1^ Values estimated from the experiments of Figure 5. ^2^ Values estimated from the experiments of Figure 7, except when indicated. ^3^ These values were estimated from inhibitor dose-response curves (Figure 6).

## Data Availability

The data presented in this study are available in the article and its supplementary material.

## Supplementary Materials

The following are available online at https://www.mdpi.com/1422-0067/22/4/1977/s1.

## Abbreviations

bis-4-NPP	Bis-4-nitrophenylphosphate
c-di-AMP	3′,5′-cyclic diadenosine monophosphate
c-di-GMP	3′,5′-cyclic diguanosine monophosphate
CpdB_Cdom	C-terminal domain of CpdB
CpdB_Ndom	N-terminal domain of CpdB
HPLC	High-performance liquid chromatography
GST	Glutathione S-transferase
EDTA	Ethylenediaminetetraacetate
pApA	5′-phosphoadenylyl-3′→5′-adenosine
PDB	Protein Data Bank
pGpG	5′-phosphoguanylyl-3′→5′-guanosine
SDS-PAGE	Sodium dodecyl sulfate polyacrylamide gel electrophoresis
Tris	Tris(hydroxymethyl)aminomethane
