# Supplementary material for: Molecular Dissection of Escherichia coli CpdB: Roles of the N Domain in Catalysis and Phosphate Inhibition, and of the C Domain in Substrate Specificity and Adenosine Inhibition
Source: Int J Mol Sci. 2021 Feb 17;22(4):1977. doi: 10.3390/ijms22041977 (PMC7922932; doi:10.3390/ijms22041977)
Supplement: Supplementary file 1 [file ijms-22-01977-s001.pdf]

Supplementary Figures and Table to:

Molecular dissection of *Escherichia coli* CpdB: roles of the N domain in catalysis and phosphate inhibition, and of the C domain in substrate specificity and adenosine inhibition

Iralis López-Villamizar, Alicia Cabezas, Rosa María Pinto, José Canales, João Meireles Ribeiro, Joaquim Rui Rodrigues, María Jesús Costas and José Carlos Cameselle

This document contains the following supplementary Figures and Table:

Figure S1. Comparison of the sequences of the precursors of mature CpdB and UshA of *Escherichia coli*.

Figure S2. Sanger sequencing of pGEX-6P-3-cpdB_Ndom and pGEX-6P-3-cpdB_Cdom constructs.

Figure S3. Sanger sequencing of mutant plasmids pGEX-6P-3-H117A_cpdB and pGEX-6P-3-Y544A_cpdB compared to the wild-type sequence in plasmid pGEX-6P-3-cpdB.

Figure S4. Expression of the recombinant proteins from plasmids pGEX-6P-3-cpdB, pGEX-6P-3-cpdB_Ndom, pGEX-6P-3-cpdB_Cdom, pGEX-6P-3-H117A_cpdB and pGEX-6P-3-Y544A_cpdB.

Table S1. PCR primers for construction of protein domains and point mutants of *E. coli* BL21 CpdB.

```
                                                   ↓ ↓
CpdB  001 MIK-----FSATLLATLIAASVNAATVD------LRIMETTDLHSNMMDFDYYKDTATEK 049
UshA  001 M-KLLQRGVALALLTTFTLASETALAYEQDKTYKITVLHTNDHHGHFWRNEYGE------ 053
          * *      . .**.*   ** .* . .      . .. *.* * .    .*

                                        ↓
CpdB  050 FGLVRTASLINDARNEVKN---SVLVDNG-DLIQGSPLADYISAKGLKAGDVHPVYKALN 105
UshA  054 YGLAAQKTLVDGIRKEVAAEGGSVLLLSGGDINTGVPESD--------LQDAEPDFRGMN 105
          .**    .*.   * **     ***.  * *.  * * .*          *  * .. .*

                    ↓↓
CpdB  106 TLDYTVGTLGNHEFNYGLDYLKNALAGAKFPYVNANVIDARTKQPMFTPYLIKDTEVVDK 165
UshA  106 LVGYDAMAIGNHEFDNPLTVLRQQEKWAKFPLLSANIYQKSTGERLFKPWALF------- 158
           . *   ..*****   *  *..    **** . **.    * . .* *  .

CpdB  166 DGKKQTLKIGYIGVVPPQIMGWDKANLSGKVTVNDITETVRKYVPEMREK-GADVVVVLA 224
UshA  159 --KRQDLKIAVIGLTTDDTAKIGNPEYFTDIEFRKPADEAKLVIQELQQTEKPDIIIAAT 216
            *.* ***  **.                .     ..  .  . *...    *...  .

          ↓                                  ↓ ↓
CpdB  225 HSGLSADP-YKVMAENSVYYLSEIPG--VNAIMFGHAH-----AVFPGKDFADIEGADIA 276
UshA  217 HMGHYDNGEHGSNAPGDVEMARALPAGSLAMIVGGHSQDTVCMAAENKKQVDYVPGTPCK 276
          * *          * . *     .*   .  *. **..     *    *    . *.

CpdB  277 KGTLNGVPAVMPGMWGDHLGVVDLQLSNNSGKWQVTQAKAEARPIYDIANKKSLAAEDSK 336
UshA  277 PDQQNGIWIVQAHEWGKYVGRADFEFRNGEMKMVNYQ-------LIPVNLKKKVTWEDGK 329
              **.  *    **  .*  * .  *.  *    *       .  .  ** .. ** *

CpdB  337 LVETLKADHDATRQFVSKPIGKSADNMYSYLALVQDDPTVQVVNNAQKAYVEHYIQGDPD 396
UshA  330 SERVLYTPEIAENQ-----------QMISLLSPFQNKGKAQL--EVKIGETNGRLEGDRD 376
              * .   *  *           .* * *.  *     *.    .       ..** *

                                                         ↓
CpdB  397 LAKLP-------VLSA-----AAPFKV--GGRKNDPASYVEVEKGQLTFRNAADLYLYPN 442
UshA  377 KVRFVQTNMGRLILAAQMDRTGADFAVMSGGGIRD-----SIEAGDISYKNVLKVQPFGN 431
            .         .*.*      * * *  **   *      .* * ....*   .  . *

CpdB  443 TLIVVKASGKEVKEWLECSAGQFNQIDPDN-TKPQSLINWDGFRTYNFDVIDGVNYQIDV 501
UshA  432 VVVYADMTGKEVIDYLTAVA----QMKPDSGAYPQ-------FANVSFVAKDG------- 473
           ..    .**** . * . *    *. **  . **       * .  *   **

                                          ↓
CpdB  502 TQPARYDGECQMVNANAERIKNLTFNGKPIDPNAMFLVATNNYRAYGGK           550
UshA  474 ------------------KLNDLKIKGEPVDPAKTYRMATLNFNATGGD           504
                            ..  *   * *.**   . .** *. * **
```

Figure S1. Comparison of the sequences of the precursors of mature CpdB and UshA of *Escherichia coli*. The blastp alignment was done in the NCBI server (http://blast.ncbi.nlm.nih.gov/Blast.cgi) using accession numbers WP_000589409 (CpdB, 647 aa) and CAA27532 (UshA, 550 aa). The black arrows signal seven amino acids which in UshA are coordinated to the metal ions of the dinuclear center. Six of them are conserved in CpdB as identities. The underlined letters correspond to amino acids which interact with substrate in the C-terminal domain of UshA [39]. The red arrows signal the three amino acids of CpdB shown in Figure 1b (main manuscript): the catalytic His^{117} in the N-terminal domain and the two aromatic residues (Tyr^{440} and Tyr^{544}) that form a stacked sandwich with the adenine ring of the docked substrate.

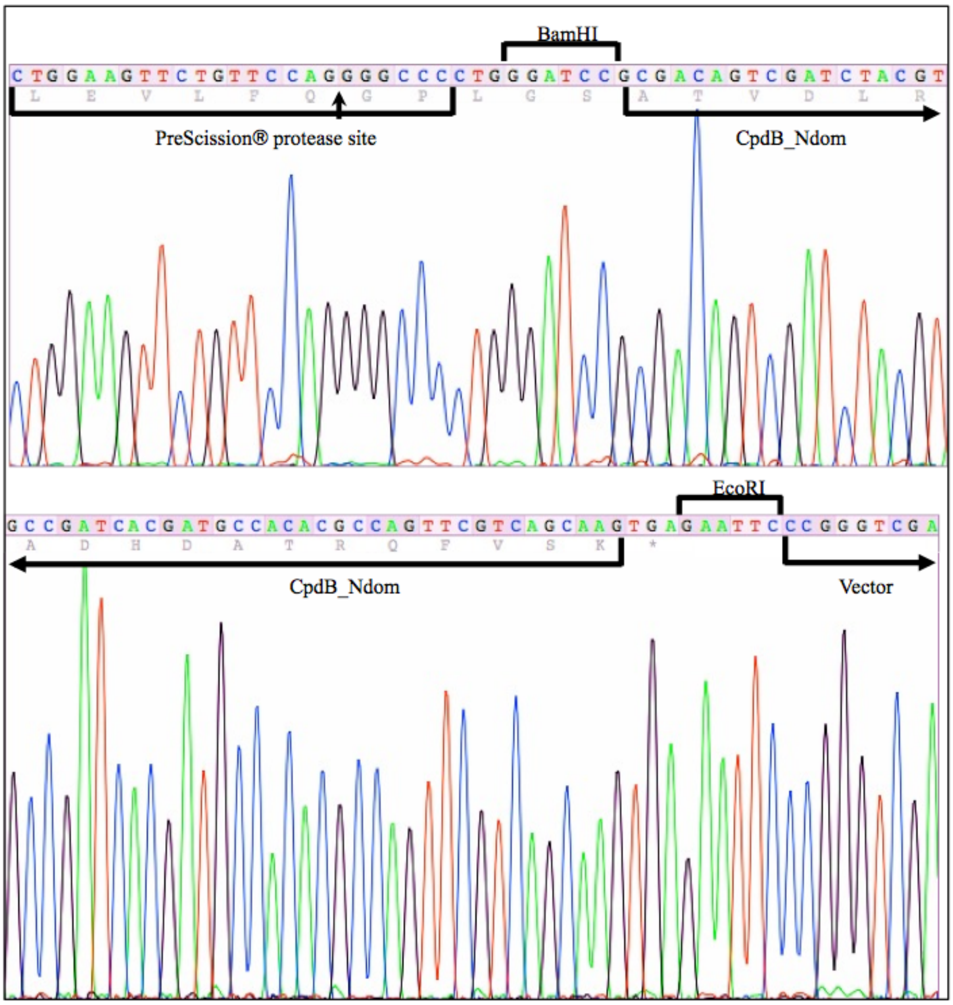

(a)

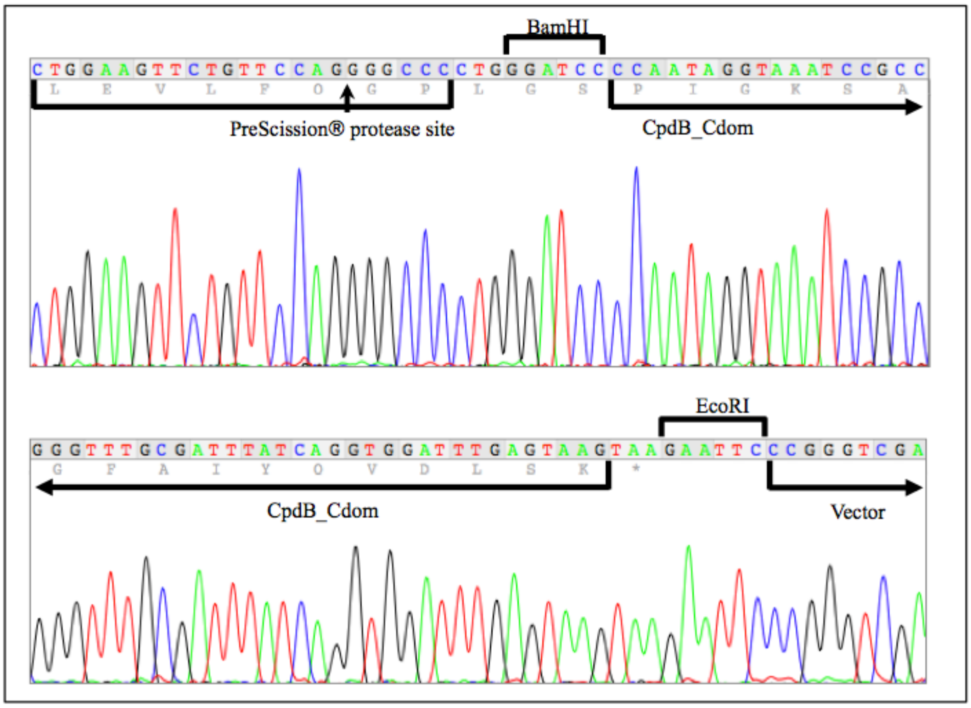

(b)

Figure S2. Sanger sequencing of (**a**) pGEX-6P-3-cpdB_Ndom and (**b**) pGEX-6P-3-cpdB_Cdom constructs.

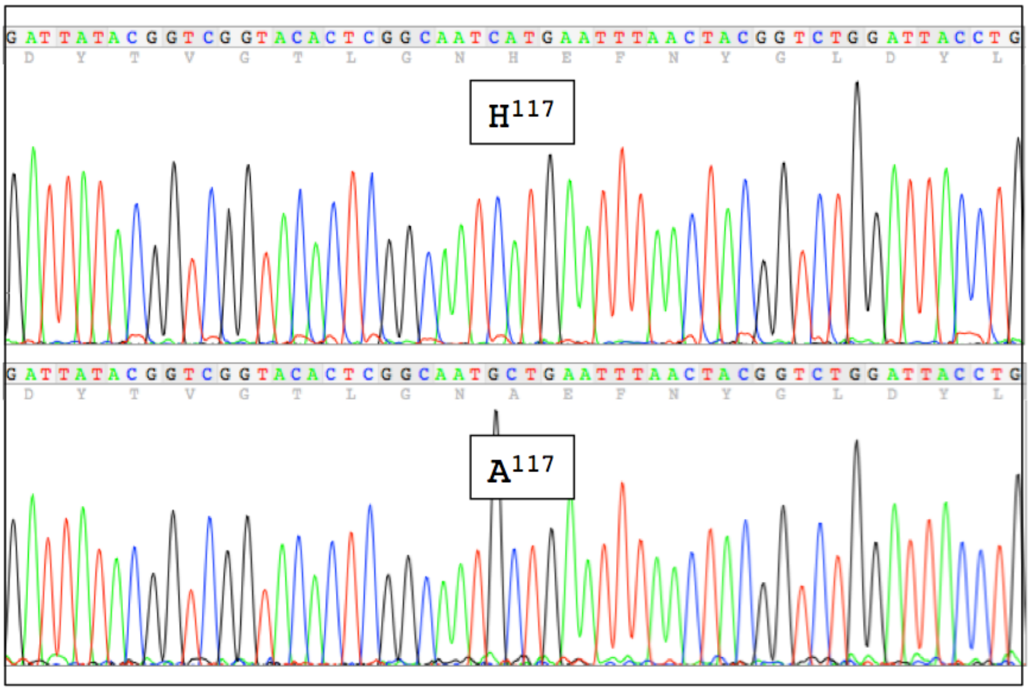

(a)

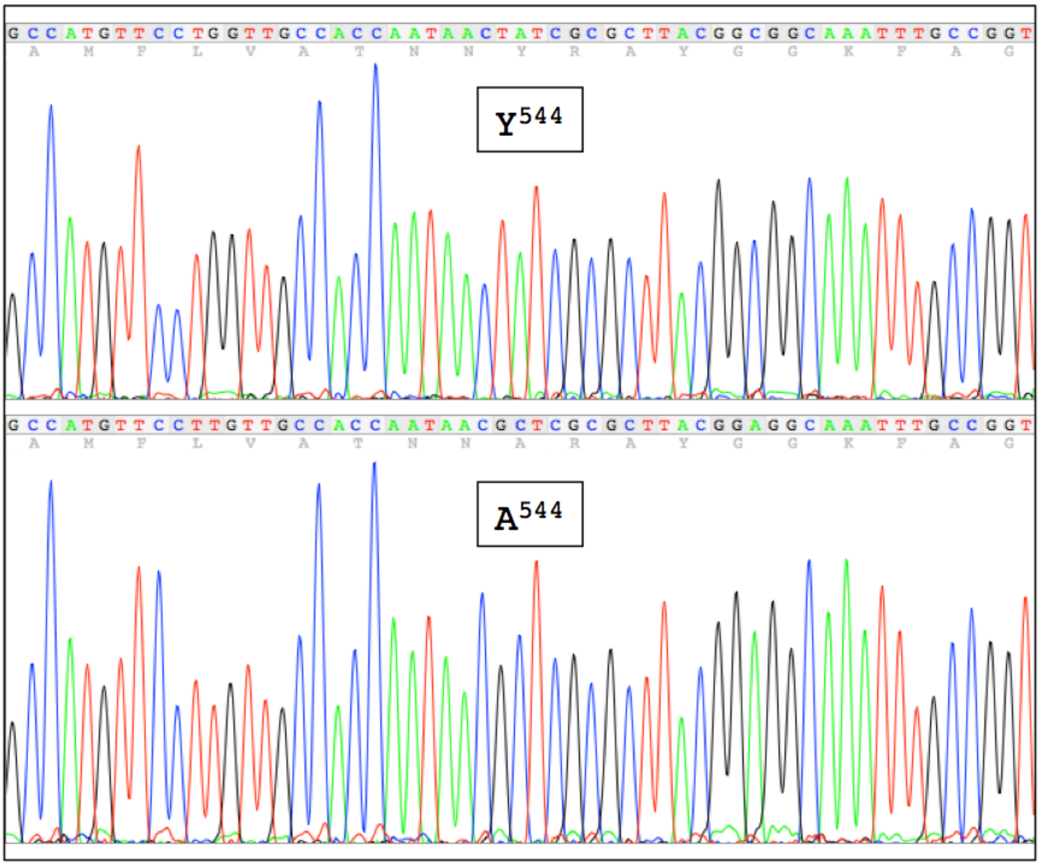

(b)

Figure S3. Sanger sequencing of mutant plasmids (**a**) pGEX-6P-3-H117A-cpdB and (**b**) pGEX-6P-3-Y544A-cpdB, both compared to the wild-type sequence in plasmid pGEX-6P-3-cpdB (upper sequence in each panel).

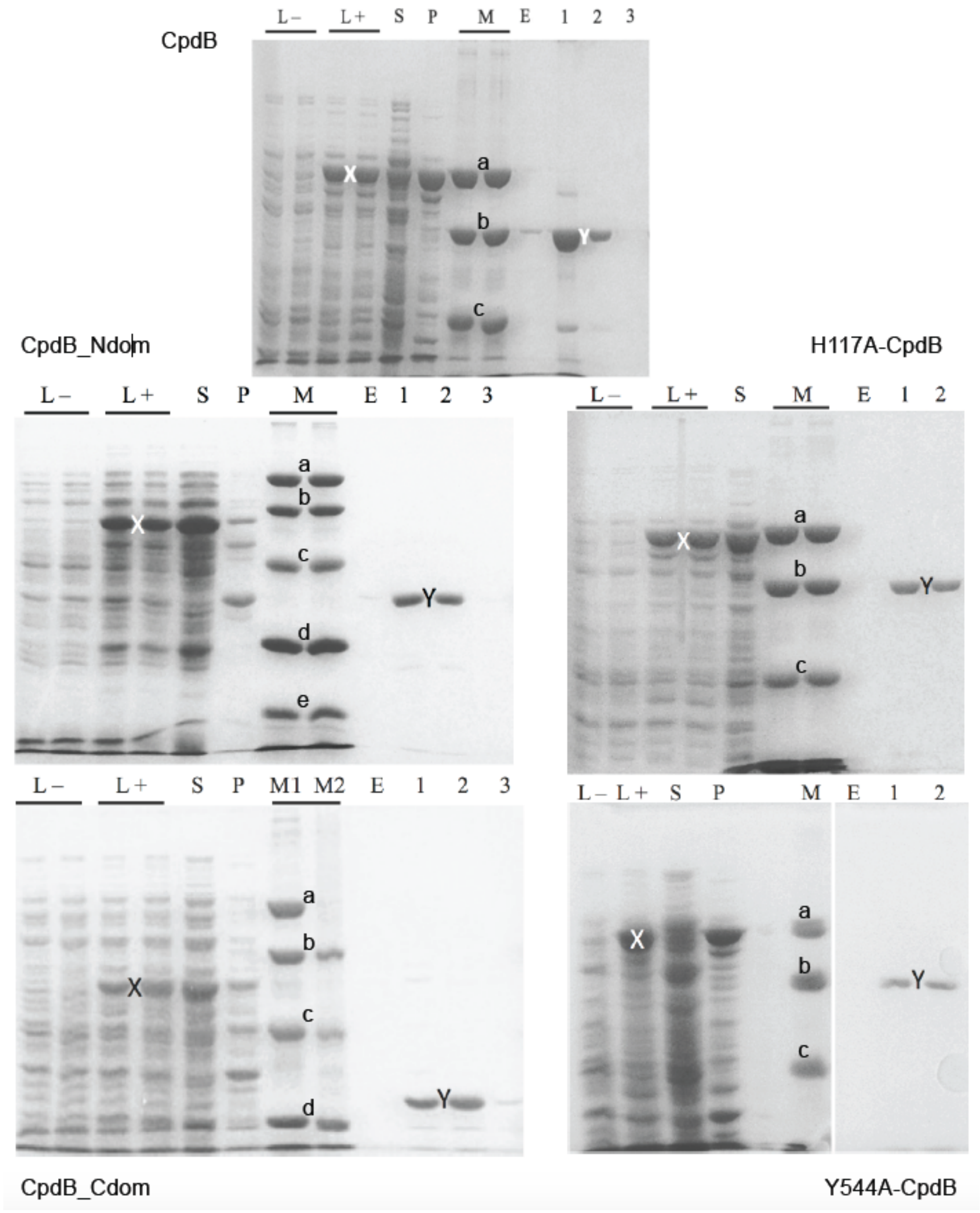

Figure S4. Expression of the recombinant proteins from plasmids pGEX-6P-3-cpdB [4], pGEX-6P-3-cpdB_Ndom, pGEX-6P-3-cpdB_Cdom, pGEX-6P-3-H117A-cpdB and pGEX-6P-3-Y544A-cpdB. L, lysate of BL21 cells transformed with the corresponding plasmid, with (+) or without (–) induction by isopropylthiogalactoside (IPTG). S and P, supernatant and precipitate of the induced cells. M, molecular weight markers: a, 97.4 kDa; b, 66.2 kDa; 45 kDa; 31 kDa; e, 21.5 kDa. E, fraction excluded from a GSH-Sepharose column after application of induce lysate supernatant. 1, 2 and 3, fractions collected from the GSH-Sepharose column after in-column removal of the GST tag of fusion proteins with PreScission protease. Letter X marks the position of the GST-fusion proteins with the following expected sizes predicted from their sequences: GST-CpdB, 95.7 kDa; GST-CpdB_Ndom, 63.4 kDa; GST-CpdB_Cdom, 59.2 kDa; GST-H117A-CpdB, 95.7 kDa; GST-Y544A-CpdB, 69.3 kDa. Letter Y marks the purified recombinant proteins devoid of the GST tag with the following expected sizes predicted from their sequences: CpdB, 69.3 kDa; CpdB_Ndom, 37.0 kDa; CpdB_Cdom, 32.8 kDa; H117A-CpdB, 95.7 kDa; Y544A-CpdB, 69.3 kDa.

Table S1. PCR primers for construction of protein domains and point mutants of *E. coli* BL21 CpdB

1	CpdB-Fow	CACTGGGGATCCGCGAC***A***GTCGATCTACGTATCATGGAAACCACTG
	Translation	A T V D L R I M E T T
2	CpdB-Rev	CTGCACGAATTCTTACTTACTCAAATCCACCTGATAAATCGCAAACCCG
	Reverse complement	cgggtttgcgatttatcaggtggatttgagtaagtaagaattcgtgcag
	Translation	G F A I Y Q V D L S K *
3	CpdB_Nterm-Rev_a	CTGCACGAATTCTCACTTGCTGACGAACTGGCGTGTGGC
	Reverse complement	gccacacgccagttcgtcagcaagtgagaattcgtgcag
	Translation	A T R Q F V S K *
4	CpdB_Cterm-Fow_a	CACCAAGGATCCCCAAT***A***GGTAAATCCGCCG
	Translation	P I G K S A
5	H117A-CpdB-Fow	GTCGGTACACTCGGCAATGCTGAATTTAACTACGGTCTGGATTAC
	Translation	V G T L G N A E F N Y G L D Y
6	Y544A-CpdB-Fow	GCCATGTTCCTTGTTGCCACCAATAACGCTCGCGCTTACGG***A***GGC
	Translation	A M F L V A T N N A R A Y G G

[1] Forward primer used to amplifiy the coding sequence of the N domain of mature CpdB (CpdB_Ndom). It includes a BamHI site (underlined) before the coding sequence, and it bears a designed silent substitution with respect to GenBank accession KP938772 (italics bold type).

[2] Reverse primer used to amplifiy the coding sequence of the C domain of mature CpdB (CpdB_Cdom). It includes an EcoRI site (underlined) to appear after the stop codon in the reverse complement.

[3] Reverse primer used to amplifiy the coding sequence of CpdB_Ndom. It includes an EcoRI site (underlined) to appear after a designed stop codon in the reverse complement.

[4] Forward primer used to amplify the coding sequence of CpdB_Cdom. It includes a BamHI site (underlined) before the coding sequence, and it bears a designed silent substitution with respect to GenBank accession KP938772 (italics bold type).

[5] Forward mutagenic primer used to used to obtain the His[117]Ala-CpdB mutant protein. An alanine codon (underlined) substituted for the histidine one. The reverse mutagenic primer (not shown) was the reverse complement of the forward one.

[6] Forward mutagenic primer used to used to obtain the Tyr[544]Ala-CpdB mutant protein. An alanine codon (underlined) substituted for the tyrosine one. In addition, the primer bears a designed silent substitution (italics bold type). The reverse mutagenic primer (not shown) was the reverse complement of the forward one.
